# A pilot study on assessing the role of intra-operative Flow 800 vascular map model in predicting onset of vasospasm following micro vascular clipping of ruptured intracranial aneurysms

**DOI:** 10.12688/f1000research.15627.1

**Published:** 2018-08-03

**Authors:** Sunil Munakomi, Deepak Poudel

**Affiliations:** 1 Neurosurgery, Nobel Teaching Hospital, Biratnagar, 0977, Nepal

**Keywords:** Aneurysm, vasospasm, ICG study, FLOW 800 study

## Abstract

**Objective**

To ascertain the predictive value of intra-operative FLOW 800 vascular map model in predicting onset of post-operative clinical vasospasm and delayed cerebral ischemia among patients undergoing micro-vascular clipping of ruptured intracranial aneurysms.

**Material and methods**

A total of 40 patients were enrolled in the study and their variables such as age, World Federation of Neurological Surgeons (WFNS) grade at presentation, Computerized Tomography (CT) Fisher grading, location of the aneurysms, and Indocyanine Green (ICG) flow status were compared and statistically analyzed along with differences in Absorption Intensities (AI) and difference in time lag values obtained from the FLOW 800 vascular map studies for predicting onset of vasospasm.

**Results**

The Receiver Operating curve (ROC) of the model for predicting post-operative vasospasm was highest (.892) for difference in the AI followed by CT Fisher grading (.778), difference in time lag (.700) and WFNS grading (.699).Analysis of variance for different variables studied in our model for predicting vasospasm was significant for all except for age (.991) and the ICG flow through the parent vessel (.079).Multivariate analysis done for predicting the vasospasm was significant for all variables except for age (.869) and ICG main flow (.196)

**Conclusion **

Our study confirmed the role of FLOW 800 study model in predicting clinical vasospasm. Inclusion of this entity would therefore help in taking timely and correct therapeutics measures to ensure better patient outcomes.

## Introduction

Progressive narrowing of vessels can occur in up to 70% of patients within 2 weeks of intra-cranial aneurysmal rupture, among which 30% develop delayed ischemic neurological deficits ^1, 2^. Although hypertension, hypervolemia and hemodilution (triple-H) therapy is commonly instituted to counteract this process, there is a paucity of evidence for recommending it for prophylactic purposes ^3^. The American Heart Association/American Stroke Association guidelines also recommend induced hypertension targeted euvolemia for managing delayed cerebral ischemia (DCI) ^4^. The World Federation of Neurological Surgeons (WFNS) grade of the patient at presentation and the location of epicenter of the subarachnoid bleed has shown to be positively correlated with the onset of vasospasm ^5, 6^. Since indocyanine green (ICG) flow studies are safe, easily applicable, readily reproducible and now routinely utilized technique during the microvascular clipping of aneurysms, addition of FLOW 800, an automated vascular map study generated by the microscope with the provision for quantitative study of flow velocities and time lag for appearance of the dye between relevant vessels, can help us segregate and outline groups at high-risk of developing post-operative vasospasm and form an evidence-based management algorithm for better therapeutic benefits and clinical outcomes.

## Methods

### Subjects and procedures

A total of 40 patients who underwent microvascular clipping for ruptured intra-cranial aneurysms in the Department of Neurosurgery at Nobel Medical College and Teaching Hospital (Biratnagar, Nepal) between January 2017 and June 2018 were enrolled in the study. Those patients who refused to participate in the study or who left the hospital against medical advice during the course of the study were excluded from the study. Moreover, exclusions were also made in extreme circumstances wherein ICG study was not possible owing to intra-operative brain swelling or inability to visualize the relevant vessels owing to small operating field or close proximities between relevant vessels rendering difficulties in correctly specifying regions of interests (ROI).

### Data acquisition

The retrospective acquisition of data of these patients, with regards to their age, Glasgow coma scale (GCS) during initial presentation, WFNS grade, computerized tomography (CT) Fisher grading ^7^, ICG flow status and FLOW 800 mapping (version 2.21) (extrapolated from a Pentero surgical microscope; Carl Zeiss Co., Germany) was conducted and the outcome in terms of occurrence of radiological and clinical vasospasm was analyzed.

To obtain ICG flow status, after the permanent clip was applied, the setting of the microscope was changed from the usual white light to the infrared mode. Next, ICG was injected and visualization was looked for within the desired vessels. This is a qualitative study, but we can record the time lag for appearance of dye. Following this, with the help of the FLOW 800 software, a vascular map was automatically generated from the IR 900 video data. There is also provisions for quantitative analysis of the flow dynamics in terms of average absorption intensity (AI) and time lag for appearance of the dye by selecting appropriate regions of interests (ROI) within the vessels.

**Figure 1.**
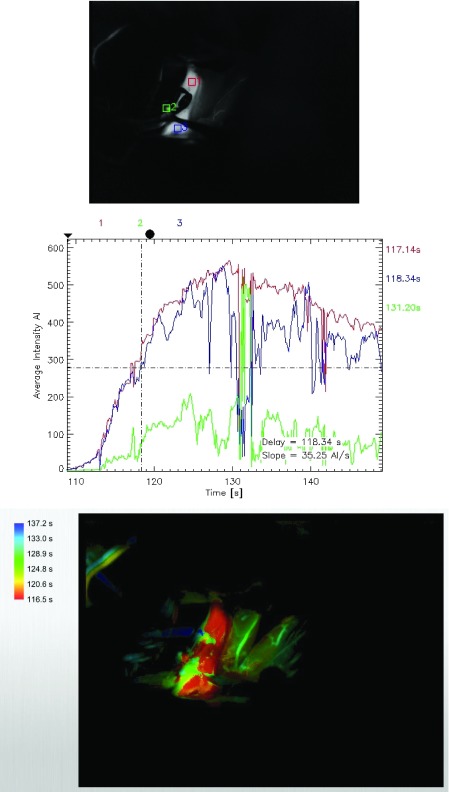
Flow 800 map in the case of a posterior communicating artery (PCOM) aneurysm showing peak flow in PCOM (green) greater than 50% of flow in the internal carotid (red) but significant (>6 seconds) delay in time lag for the flow.

 In our study model of FLOW 800 mapping, the lower normal limit for normal difference in average absorption intensity (DfAI) between the parent and the branching vessel was taken at 50%. Similarly, the maximum upper limit for time-lag for appearance of the flow between the parent and the branching vessel was kept at 6 seconds ( Figure 1– Figure 4). This time limit was extrapolated from the pooled results of ICG and FLOW 800 studies in patients who did not show any vasospasm in the post-operative period. The average intensity was chosen owing to its easy applicability, calculation and easy reproducibility, thereby minimizing scoring bias. Currently, there is a paucity in the literature with regards to normal values for AIs and time-lag for specific vessels following FLOW 800 study so as to form a specific reference standard. Values from these studies were compared to clinical findings and CT images, which served as the surrogate marker for vasospasm.

**Figure 2.**
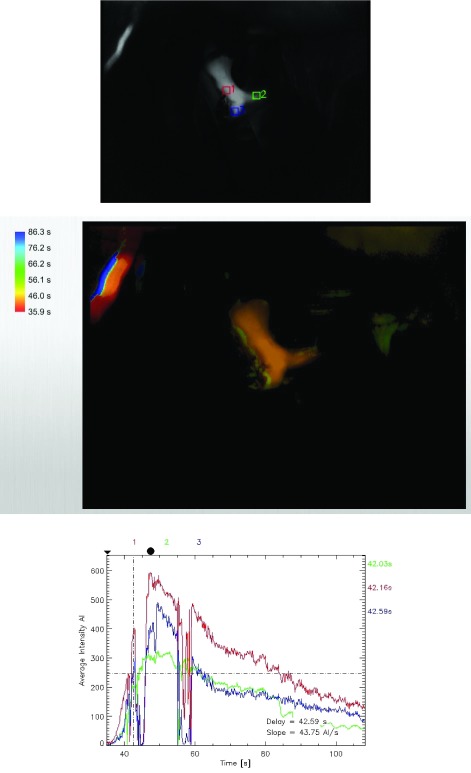
Flow 800 map in the case of an anterior communicating artery aneurysm showing flow in the A1 segment (green) at 50% of the parent internal carotid artery (red).

**Figure 3.**
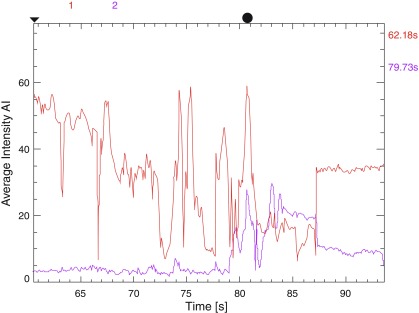
Flow map in the same patient as in Figure 2 showing flow discrepancy of almost 50% between the two distal A2’s as well as significant time delay in flow through them.

**Figure 4.**
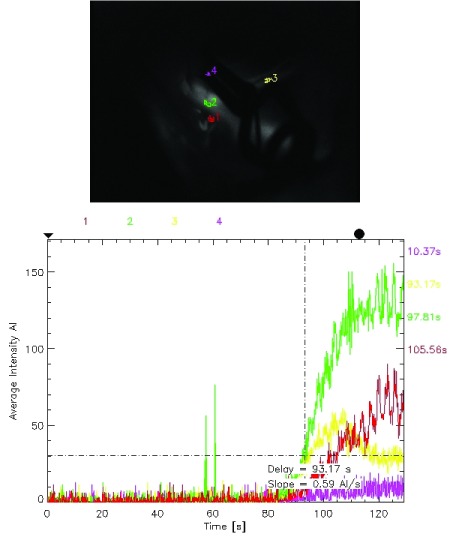
Flow map in another anterior communicating artery aneurysm also revealing flow in A2’s (yellow) less than 50% of that in the A1 (green).

**Figure 5.**
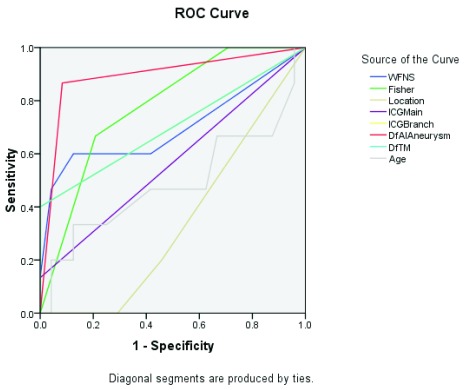
Receiver operating curve for different variables in the study for predicting vasospasm.

To minimize study bias and the post hoc effects, the study was blinded among the investigators who studied the clinical and radiological outcomes from those who analyzed the ICG and FLOW 800 vascular studies. All patients received an intravenous ICG bolus of 25 mg dissolved in 5 ml of 0.9% saline for the study. A Pentero surgical microscope (Carl Zeiss Co., Germany) was utilized for studying ICG as well as FLOW 800 vascular studies.

### Post-operative care

Post-operatively, patients were given 60 mg (oral or via naso-gastric feeding tube) nimodipine every 4 hours and fluid management was titrated to achieve a central venous pressure of 8–12 mmHg, a hematocrit of 30–35% and a mean arterial pressure (MAP) of more than 20 mmHg greater than the preoperative MAP value. Clinical (presence of new onset neurological deficits in the post-operative period) and radiological (presence of features of ischemia or infarction) evidence of vasospasm were stringently assessed and recorded in the post-operative period.

### Ethical considerations

The study was approved by the Institutional Review Committee (IRC) of Nobel Medical College and Teaching Hospital (NMCTH) (approval number 134/2018). Written informed consent was taken from the relatives or next of kin of the patients (owing to the poor neurological status of the patients and the emergent need for operative management) for their inclusions in the study and usage of their relevant clinical data for resource measures.

### Data analysis

Data were recruited and analyzed using the SPSS version 16 software. Statistical analysis was done utilizing receiver operating curve (ROC) with area under curve (AUC) values, Analysis of variance (ANOVA) and multivariate logistic regression analysis along with logistic coefficient curve study among the considered variables applying vasospasm as the final outcome. No post hoc analysis was done beyond those factors pertaining to our study model.

## Results

The incidence of clinical vasospasm and delayed cerebral ischemia was 40% in this study. CT Fisher grade 3 was seen in 42.5% of cases and grade 4 in 37.5% of cases. The incidence of anterior communicating artery aneurysm was observed in 62.5% of cases.

The receiver operating curve (ROC) of the model for predicting post-operative vasospasm was highest (area under the curve (AUC)=0.892) for difference in the AI of FLOW 800 study followed by CT Fisher grading (AUC=0.778), difference in time lag in FLOW 800 (AUC=0.700) and WFNS grading (AUC=0.699) ( Figure 5 and Table 1) thereby verifying the aim of our study for its routine inclusion as an intra-operative adjunct to ICG flow study.

**Table 1.** Area under curve values for different variables in predicting vasospasm.

Test result variable(s)	Area	Std. error	Asymptotic significance	Asymptotic 95% confidence interval	
				Lower bound	Upper bound
WFNS	0.699	0.097	0.039	0.508	0.889
Fisher	0.778	0.075	0.004	0.632	0.924
Location	0.342	0.086	0.100	0.173	0.511
ICG main	0.567	0.098	0.488	0.375	0.758
ICG branch	0.700	0.094	0.038	0.516	0.884
Difference (AI)	0.892	0.061	0.000	0.772	1.012
Difference (time)	0.700	0.094	0.038	0.516	0.884
Age	0.490	0.104	0.920	0.286	0.695

WFNS, World Federation of Neurological Surgeons; ICG, indocyanine green; AI, absorption intensity.

ANOVA for variables studied in our model for predicting vasospasm was significant for WFNS grade, CT Fisher grade, location of aneurysms, ICG flow through branching vessels, difference in flow velocities (DfAI) and time lag in dye appearance (DfTM) but not for age of the patients (P=0.991) and the ICG flow through the parent vessel (P=0.079) ( Table 2). Multivariate analysis done for predicting the vasospasm was significant for all variables except for age (P=0.869) and ICG main flow (P=0.196) ( Table 3).

**Table 2.** Results of analysis of variance for different variables in the study with regards to predicting vasospasm.

Variable	Comparison	Sum of squares	df	Mean square	F	Sig.
Age	Between groups	0.017	1	0.017	0.000	0.991
	Within groups	5235.583	38	137.779		
WFNS	Between groups	16.017	1	16.017	11.359	0.002
	Within groups	53.583	38	1.410		
Fisher	Between groups	5.192	1	5.192	12.667	0.001
	Within groups	15.167	37	0.410		
Location	Between groups	4.267	1	4.267	4.402	0.043
	Within groups	36.833	38	0.969		
ICG Main	Between groups	0.150	1	0.150	3.257	0.079
	Within groups	1.750	38	0.046		
ICG Branch	Between groups	1.350	1	1.350	13.680	0.001
	Within groups	3.750	38	0.099		
Difference (AI)	Between groups	6.017	1	6.017	63.805	0.000
	Within groups	3.583	38	0.094		
Difference (time)	Between groups	1.350	1	1.350	13.680	0.001
	Within groups	3.750	38	0.099		

df, degrees of freedom; WFNS, World Federation of Neurological Surgeons; ICG, indocyanine green; AI, absorption intensities.

**Table 3.** Results of multivariate analysis of various variables for predicting vasospasm.

Source	Dependent variable	Type III sum of squares	df	Mean square	F	Sig.
Corrected model	Age	39.626	2	19.813	0.141	0.869
	WFNS	14.407	2	7.203	5.036	0.012
	Fisher	5.207	2	2.604	6.186	0.005
	Location	6.574	2	3.287	3.440	0.043
	ICG main	0.164	2	0.082	1.704	0.196
	ICG branch	1.477	2	0.738	7.385	0.002
	Difference (AI)	6.043	2	3.021	34.121	0.000
	Difference (time)	1.477	2	0.738	7.385	0.002

df, degrees of freedom; WFNS, World Federation of Neurological Surgeons; ICG, indocyanine green; AI, absorption intensities.

**Figure 6.**
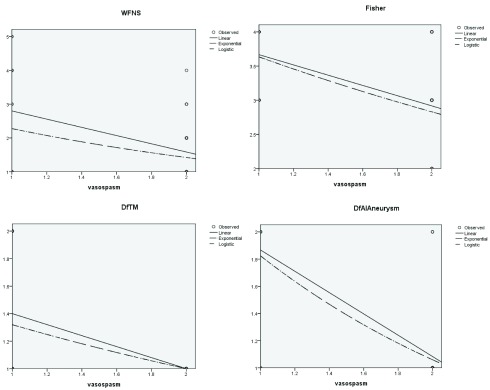
Logistic correlation curve of various variables with onset of vasospasm. The specific variable is represented in in the ‘x’ axis and risk of vasospasm in the ‘y’ axis. DfAI, difference in average absorption intensity between parent and branching vessels; DfTM, difference in time lag for appearance of dye between parent and branching vessels.

**Figure 7.**
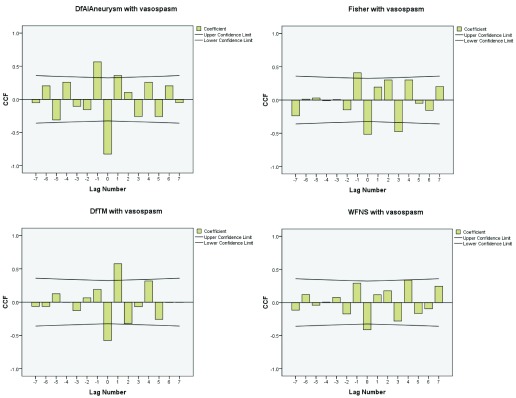
Logistic coefficient curve of various factors with onset of vasospasm. DfAI, difference in average absorption intensity between parent and branching vessels; DfTM, difference in time lag for appearance of dye between parent and branching vessels.

The correlation and the coefficient curves between relevant variables and onset of post-operative vasospasm have been shown in the Figure 6 and Figure 7, which are also positively correlated with WFNS, CT Fisher grade, difference in velocities (DfAI) and time lag (DfTM) obtained from the FLOW 800 software in positively predicting the onset of post-operative vasospasm.

Demographic information and the results of each diagnostic technique performed for each patient Click here for additional data file. Copyright: © 2018 Munakomi S and Poudel D 2018 Data associated with the article are available under the terms of the Creative Commons Zero "No rights reserved" data waiver (CC0 1.0 Public domain dedication).

## Discussion

Cerebral vasospasm has surpassed re-bleeding as the main cause of death and major disability among patients with ruptured intracranial aneurysms ^9^. Inadvertent clipping of the parent vessels, its branches or the perforators causing compromised blood flow has been seen in 12–21% of cases, with subsequent occurrence of significant vasospasm in up to 10% of these cases ^10, 11^. Cerebral vasospasm still leads to mortality in 7% of patients, and to severe disability in another 7% of cases, even in experienced hands and the best neurosurgical care ^12– 14^. Visual inspection alone does not verify perfect placement of the clips and guarantee the patency of the relevant vessels ^10, 15^.

In this aspect, intraoperative angiography is still the gold standard in confirming the patency of the parent and their branching vessels along with their relevant perforators ^16, 17^. Intra-operative angiography has facilitated clip readjustment in almost 44% of cases. However, technical complexities, the risk of radiation hazards and major neurological complications (0.4–2.6%) preclude its frequent intra-operative application ^18^. The application of intraoperative angiography also prolongs the operative time by almost 40 minutes ^19^.

Microscope-integrated ICG angiography has shown to be a valuable alternative for assessing real-time intra-operative vascular mapping with minimal risks and hazards compared to intra-operative digital subtraction angiography ^20, 21^. ICG is a near-infrared (NIR) fluorescent dye; its absorption and emission peaks (805 and 835 nm, respectively) are ideally suited for usage in vascular studies since confounding absorption from other endogenous chromophores are minimal within these ranges ^22^. The dye remains confined within the vascular compartment after binding to specific plasma proteins. The operating microscope can be integrated with a laser light source (wavelength within the ICG absorption band) and a camera that is capable of exciting, visualizing and subsequently transforming acquired ICG images into real-time vascular road maps ^21, 23– 25^.

FLOW 800 is microscope-integrated software capable of automatically reconstructing time-resolved quantitative analysis of ICG angiography studies. The resulting data can be displayed and stored as either time-to-arrival maps or time-intensity curves specific for selected regions of interests (ROI) ^26^. The software reproduces a map graded by the averages of the AIs and the time lag for the same based on time to half maximal fluorescence within the selected ROIs ^27^. ICG angiography has complication rate of less than 0.1% ^28, 29^. The discordance between ICG and intraoperative digital subtraction angiography (DSA) in previous series were reported to be in the range of 10–20% ^30– 32^. A recent study has shown to be correlating with post-operative DSA findings in almost 97% of cases ^23^.

The main limitation of ICG angiography is that it can only study the vascular flow within the field of the operating microscope. Furthermore, blood clots, brain tissue and sometimes applied aneurysm clips can also preclude the proper visualization of the vessels, thereby requiring further adjustments of the microscope. The image quality can also be hindered by calcifications, atherosclerotic plaques or thromboses within the aneurysm ^33^.

The limitations of having that microscope in the direct line-of-sight of the region of interest can be counteracted by simultaneous use of an endoscope ^25, 34^. A recent study suggested the use of intra-arterial ICG at a reduced dosage for better image quality with minimized time-lag between successive studies, owing to its rapid clearance, unlike intravenous studies wherein the ICG remains for around 10 minutes ^35^. Micro-vascular Doppler study is a simple and readily applicable alternative for assessing the vascular patency ^36^. A micro-Doppler study, however, lacks quantitative assessment and is also highly influenced on the insonation angles during its placement by the operator ^33, 36^. The sensitivity and specificity for determining the accurate flow are limited to 85–90% ^37, 38^. Intraoperative alterations, such as brain shift following retractor removal, probable induced late mechanical thrombosis and sometimes the Coanda effect induced by clips are not detectable by Doppler study ^19, 39^. Other more recent advancements involve somato-sensory evoked potentials (SSEPs) ^40, 41^. However, they have limitations in predicting ischemia outside the perimeter of the corticospinal tract ^25^. The ‘ultimate, all-in-one’ diagnostic tool has not yet been designed ^42^.

Prophylactic hypervolaemic therapy is unlikely to confer any additional benefit in minimizing vasospasm ^42^. Treatment with triple-H therapy causes complications in 10–20% of patients, with pulmonary edema the most common adverse effect ^13, 43^. Moreover, there can be exacerbation of cerebral edema and an added risk of bleeding from unsecured and sometimes hemorrhagic infarctions in ischemic regions ^9^.

The advantage of using FLOW 800 intra-operatively is that it facilitates the timely undertaking of corrective measures, such as readjustment of the clips. Calcium overloading, which triggers phosphorylation of the contractile proteins of the arterial smooth muscles thereby leading to vasospasm, can be minimized by using topical sodium nitroprusside (SNP) ^44, 45^. Moreover, complications such as pulmonary edema due to aggressive medical management can be minimized by placement of a swan gauge catheter to keep the pulmonary capillary wedge pressure below the colloid oncotic pressure (COP) ^46^. A serial bedside transcranial Doppler (TCD) study can be utilized in these patients to assess changes in the flow velocities and accordingly modifying the treatment algorithm ^47^. Moreover, relevant rescue interventions, such as hemodynamic augmentation or intra-arterial vascular manipulations can be timely initiated for prevention as well as management of refractory vasospasms ^48^.

There are some limitations in our study. The results of our study were derived from sample size of only 40 patients, and therefore needs further confirmation from multi-centric randomized control trials with the inclusion of larger cohorts. There is also a prerequisite for an ICG-integrated operating microscope with added facilities for FLOW 800 vascular study. In cases of the repeated use of ICG, there may be bias in the extrapolated results of FLOW 800 owing to false fluorescence from the retained dye. There is also provision for inter-rater bias when selecting the appropriate ROIs among vessels in close proximities, thereby increased tendency for false results. However, this improves with practice since there is not a steep learning curve.

## Conclusion

The addition of FLOW 800 quantitative mapping following a routinely performed ICG study can precisely help determine patients at high risk of post-operative vasospasm. Timely actions, such as readjusting clips, the local administration of drugs or aggressive medical or interventional management can be undertaken. Additional measures, such as the placement of a swan gauge catheter to minimize complications and TCD for continuous monitoring of these patients can be utilized for better clinical outcomes. Further studies are recommended for confirming the role of FLOW 800 software as a valuable adjunct to intra-operative ICG vascular studies.

## Data availability

The data referenced by this article are under copyright with the following copyright statement: Copyright: © 2018 Munakomi S and Poudel D

Data associated with the article are available under the terms of the Creative Commons Zero "No rights reserved" data waiver (CC0 1.0 Public domain dedication). 

Dataset 1. Demographic information and the results of each diagnostic technique performed for each patient. https://doi.org/10.5256/f1000research.15627.d212875 ^8^.
