# Supplementary material for: A pilot study on assessing the role of intra-operative Flow 800 vascular map model in predicting onset of vasospasm following micro vascular clipping of ruptured intracranial aneurysms
Source: F1000Res. 2018 Aug 3;7:1188. [Version 1] doi: 10.12688/f1000research.15627.1 (PMC6124383; doi:10.12688/f1000research.15627.1)
Supplement: Peer review history

# Peer review: Referee response for version 1

**DOI:** 10.5256/f1000research.17049.r36816

**Published:** 2018-09-13

**Authors:** Amit Agrawal

**Affiliations:** 1 Department of Neurosurgery, Narayana Medical College and Hospital, Nellore, Andhra Pradesh, India

In present study the authors share there preliminary experience with automated vascular mapping to identify sub-group of patient who are high-risk of developing post-operative vasospasm following aneurysmal surgery for spontaneous subarachnoid hemorrhage. It will be beneficial if an evidence based algorithm can be generated and can be used to improve clinical outcomes. Although the article is well written, however the study includes a small number of patients; data were acquired retrospectively and does not include all consecutive cases. As authors have suggested transcranial Doppler study (TCD) at frequent interval can be used as a non-invasive tool to determine the velocity in the cerebral vessels and can be correlated with FLOW 800 studies. Additionally we need to determine the cost-effectiveness of the procedures particularly in settings where the resources are limited.  To better understand the benefits of FLOW 800 we need a larger and long term experience.

I have read this submission. I believe that I have an appropriate level of expertise to confirm that it is of an acceptable scientific standard.

# Peer review: Referee response for version 1

**DOI:** 10.5256/f1000research.17049.r36812

**Published:** 2018-09-04

**Authors:** Lekhjung Thapa

**Affiliations:** 1 National Institute of Neurological and Allied Sciences, Kathmandu, Nepal

I consider this paper as an excellent report and is an important addition to the existing information on the subject. I personally adored the concept of exploring better markers to identify patients of ruptured intracranial aneurysm at high risk of postoperative clinical vasospasm and delayed cerebral ischemia and the promising findings of components of ICG flow status as one of the important components and predictors as tested by various statistical methods. I hope the author intend to study the concept in larger number of patients with ruptured intracranial aneurysm.

 In addition I would like to add few comments: 

**Abstract: **

- In the methodology section, the authors can mention ….. (Last line) ….predicting onset of vasospasm and delayed cerebral ischemia, as per the objective of the study.
- In the conclusion section, it may be better to replace word “confirmed” in “our study **confirmed**…..” I feel this study has found/demonstrated the role FLOW 800…… rather than establishing it.

**Introduction:**

1. This section is well written with the emphasis on the potential role of information generated by FLOW 800 vascular mapping. However the last sentence appears to be long. The authors could break up the sentences with pertinent references so that it is easily understood.

- “Indocyanine green (ICG) flow studies are safe, easily applicable, readily reproducible and now routinely used during the microvascular clipping of aneurysms. [Reference?]
- A note on current status of ICG studies in predicting vasospasm would be better here.

**Methods:**

- The author mentions CLINICAL VASOSPASM in introduction and in methodology section “radiological vasospasm” is also mentioned. Kindly elaborate.
- It is wiser to mention why ANOVA was chosen when multivariate analysis is also done in this case. Why were these tests chosen and how would they help in interpretation in this test.

**Discussion:**

- I think the authors should discuss more about their findings with appropriate reference rather than reviewing about the machine and methods.

**Conclusion:**

- With the limitation explained by the authors, this study obviously may not **precisely** identify patients at high risk of post-operative vasospasm, so it may be prudent to conclude by remaining confined to the objective and the the relevant result of the study.

I have read this submission. I believe that I have an appropriate level of expertise to confirm that it is of an acceptable scientific standard.

# Peer review: Referee response for version 1

**DOI:** 10.5256/f1000research.17049.r37112

**Published:** 2018-08-29

**Authors:** Guru Dutta Satyarthee

**Affiliations:** 1 All India Institute of Medical Sciences, New Delhi, Delhi, India

Intra-operative Neurosurgical Armamentarium to deal with vasospasm development following aneurysmal subarachnoid hemorrhage.

 Authors reported utility of intra-operative FLOW 800 vascular map model with indigo cyanine [ICG] fluoroscopic angiographic images obtained with microscope with appropriate facility in predicting onset of clinical cerebral vasospasm during post-operative period and delayed cerebral ischemic deficit in a cohort of forty patients suffering with aneurysmal subarachnoid hemorrhage and managed microsurgical clipping. Factors predicting risk of vasospasm onset included parameters obtained with FLOW 800 vascular map model like the difference in the absorption intensities and time lag.

 However, major disadvantages of the vascular map model include the cost factor as advanced microscope along with appropriate software is a pre-requisite, in addition to the availability of dye, and part of vessel evaluated is very limited to microscope field with direct line of-sight of target vessels but affected by obstructed microscope field by presence of blood clots and aneurysm clips. The anatomical factor of vessel and aneurysm can also impair image quality of atherosclerotic plaques, aneurysmal sac calcifications or thrombosis. Authors rightly advocated vascular model with ICG angiography can act as one of adjunct for aneurysm surgery in neurosurgical armamentarium besides vascular Doppler, endoscope, with capability of predicting vasospasm and appropriate measure can be applied right from intraoperative period like drainage of hematoma, installation of papavarin, Omaya reservoir and installation of papavarin in the post-operative period besides stellate ganglion block, and hemodynamic interventions can definitely aid in improving outcome. However, the size of the cohort sample was modest.

I have read this submission. I believe that I have an appropriate level of expertise to confirm that it is of an acceptable scientific standard.
